# Towards an arthritis flare-responsive drug delivery system

**DOI:** 10.1038/s41467-018-03691-1

**Published:** 2018-04-03

**Authors:** Nitin Joshi, Jing Yan, Seth Levy, Sachin Bhagchandani, Kai V. Slaughter, Nicholas E. Sherman, Julian Amirault, Yufeng Wang, Logan Riegel, Xueyin He, Tan Shi Rui, Michael Valic, Praveen K. Vemula, Oscar R. Miranda, Oren Levy, Ellen M. Gravallese, Antonios O. Aliprantis, Joerg Ermann, Jeffrey M. Karp

**Affiliations:** 1 Center for Nanomedicine and Division of Engineering in Medicine, Department of Medicine, Brigham and Women’s Hospital, Boston, MA 02115 USA; 2 Harvard–Massachusetts Institute of Technology Division of Health Sciences and Technology, Massachusetts Institute of Technology, Cambridge, MA 02139 USA; 3 Harvard Medical School, Boston, MA 02115 USA; 4 Division of Rheumatology, Immunology and Allergy, Department of Medicine, Brigham and Women’s Hospital, Boston, MA 02115 USA; 5 Division of Rheumatology, Department of Medicine, University of Massachusetts Medical School, Worcester, MA 01605 USA; 6 Present Address: Institute for Stem Cell Biology and Regenerative Medicine (inStem), UAS-GKVK post, Bellary Road, Bangalore, 560065 India; 7 Present Address: Merck Research Laboratories, 33 Ave Louis Pasteur, Boston, MA 02115 USA

## Abstract

Local delivery of therapeutics for the treatment of inflammatory arthritis (IA) is limited by short intra-articular half-lives. Since IA severity often fluctuates over time, a local drug delivery method that titrates drug release to arthritis activity would represent an attractive paradigm in IA therapy. Here we report the development of a hydrogel platform that exhibits disassembly and drug release controlled by the concentration of enzymes expressed during arthritis flares. In vitro, hydrogel loaded with triamcinolone acetonide (TA) releases drug on-demand upon exposure to enzymes or synovial fluid from patients with rheumatoid arthritis. In arthritic mice, hydrogel loaded with a fluorescent dye demonstrates flare-dependent disassembly measured as loss of fluorescence. Moreover, a single dose of TA-loaded hydrogel but not the equivalent dose of locally injected free TA reduces arthritis activity in the injected paw. Together, our data suggest flare-responsive hydrogel as a promising next-generation drug delivery approach for the treatment of IA.

## Abstract (web summary)

The treatment of inflammatory arthritis by local delivery of therapeutics is limited by short half-lives of drugs. Here the authors demonstrate a hydrogel platform that titrates drug release to arthritis activity.

## Introduction

Inflammatory arthritis (IA) encompasses a spectrum of inflammatory arthropathies affecting individual joints (monoarthritis), a few joints (oligoarthritis), or many joints (polyarthritis). In polyarthritides-like rheumatoid arthritis, systemic therapy is generally indicated and appropriate. However, in situations where only one or a few joints are involved, local therapy with intra-articular injections may offer distinct advantages over systemic therapy by increasing the drug bioavailability locally and reducing the potential for drug-induced systemic toxicity. Unfortunately, drugs injected into joints are often cleared very rapidly (*t*_1/2_ ∼0.1–6 h)^1,2^, which severely limits the feasibility of this approach. Multiple platforms have been developed to increase the joint residence time of drugs after intra-articular injection, including liposomes^3,4^, solid lipid nanoparticles^5^, micelles^6^, polymeric microparticles/nanoparticles^7–9^, and hydrogels^10,11^. Such systems have been shown to increase intra-articular drug half-lives by at least 10–30-fold in preclinical models^2^. However, none of the formulations have advanced past clinical trials for IA.

IA often exhibits variable disease activity over time with exacerbations (flares) and periods of low disease activity. Previously described intra-articular drug delivery systems provide sustained drug release irrespective of disease activity. This likely results in sub- or supra-therapeutic drug levels locally during periods of high or low disease activity, respectively. A flare-responsive drug delivery system would titrate drug release to match the disease activity, resulting in optimal therapeutic efficacy. It would also minimize unnecessary drug release during periods of low disease activity, thereby prolonging the joint residence time of the injected drug depot and hence the duration of the therapeutic effect.

We have developed an arthritis flare-responsive hydrogel platform by self-assembling a small-molecule amphiphile, triglycerol monostearate (TG-18). This compound was identified in a screen of the Food and Drug Administration’s (FDA’s) list of generally recognized as safe (GRAS) compounds looking for hydrogelators that can encapsulate a wide range of therapeutic agents and have an enzyme-labile ester linkage to facilitate hydrogel disassembly and drug release in response to enzymatic activities present in inflammatory milieus^12^. As a GRAS agent, TG-18 is relatively inexpensive and commercially available in large quantities at high purity (Good Manufacturing Practice Grade or Food Grade). In this study, we loaded TG-18 hydrogel with the corticosteroid triamcinolone acetonide (TA) as a model drug and demonstrated drug release in response to arthritis-related enzyme activities in vitro. Using the K/BxN mouse model of IA^13,14^ we showed that TG-18 hydrogel disassembly and hence the release of an encapsulated dye correlate with the severity of the arthritis flare in vivo. A single dose of TA-loaded hydrogel but not the equivalent dose of locally injected free TA reduced arthritis activity in the injected paw. Our study provides proof of concept for TG-18 hydrogel as a potential next-generation drug delivery platform for the treatment of IA. We demonstrate that flare-responsive intra-articular drug release, i.e. the matching of drug release to current disease activity, is feasible and deserves further exploration.

## Results

### TG-18 self-assembly to hydrogel and encapsulation of TA

**Fig. 1:**
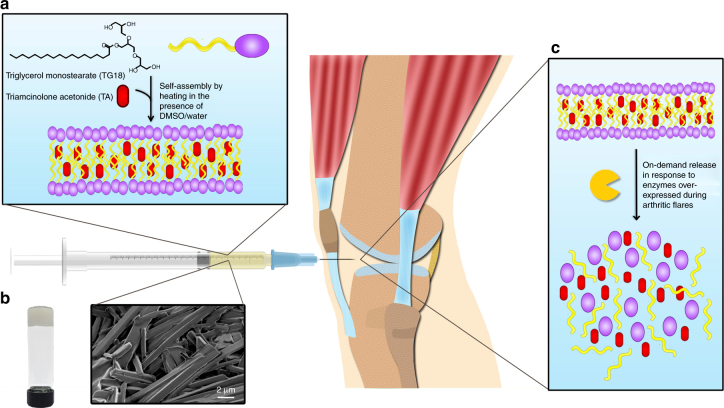
Triglycerol monostearate (TG-18) self-assembles to form arthritis flare-responsive hydrogel. **a** Schematic showing self-assembly of TG-18 to form hydrogel and encapsulation of triamcinolone acetonide (TA). TG-18 dissolves by heating in DMSO/water and self-assembles into lamellar structures upon cooling. Drugs like triamcinolone acetonide (TA) can be encapsulated within the hydrophobic core of the lamellae. **b** High-resolution scanning electron microscopy (HR-SEM) of TA-loaded TG-18 hydrogels. TG-18 lamellar structures extend to form higher order fibrous assemblies. The entangled fibers form the bulk injectable hydrogel that can be administered via intra-articular injection. **c** Disassembly of TG-18 hydrogel in response to flare-associated enzymes, including MMPs that are present in the inflamed joint

TG-18 comprises a polyhydroxyl hydrophilic head group and a polymethylene hydrophobic tail (Fig. 1a), and has a Krafft point of approximately 55–60 °C in a dimethyl sulfoxide (DMSO)/water mixture. Therefore, TG-18 heated in DMSO/water to 55–60 °C dissolves due to micelle formation, resulting in a clear solution. Upon cooling, the solution forms a solid hydrogel comprising self-assembled fibrous structures with interdigitated bilayers and extended micelles^15^ (Fig. 1a). During self-assembly, hydrophobic drugs can be encapsulated into the hydrophobic core of the lamellar fibers of the hydrogel (Fig. 1a). The drug-loaded bulk hydrogel is injectable and can be administered as an intra-articular injection. Within joints, enzymes overexpressed during arthritis flare can disassemble the hydrogel, resulting in flare-responsive drug release (Fig. 1c).

We encapsulated TA into TG-18 hydrogel (10% w/v) and were able to achieve loading efficiency as high as 40% (w/w). Higher order fibrous assemblies of TA-loaded hydrogel were observed with high-resolution scanning electron microscopy (HR-SEM) (Fig. 1b). To determine the stability of TA encapsulation into TG-18 hydrogel, we evaluated the in vitro release of TA in phosphate-buffered saline (PBS, pH 7.4) at 37 °C comparing hydrogel formulations generated with the same amount of TG-18 (10% w/v), but loaded with different concentrations of TA. We observed a dose–response relationship between the TA concentration and its encapsulation stability in the hydrogel (Supplementary Fig. 1). Hydrogels loaded with 10, 20, or 30 mg/ml TA showed excellent long-term stability in PBS with less that 35% cumulative release of TA over a period of 30 days. Hydrogel containing 40 mg/ml TA showed 45% cumulative release over 30 days. We did not observe substantial burst release within the first few hours for any of the formulations. HR-SEM imaging revealed patches of non-fibrous precipitates in hydrogels loaded with 30 mg/ml and even more so with 40 mg/ml TA (Supplementary Fig. 2), suggesting that that TA at high concentrations interferes with TG-18 self-assembly. Based on these data we chose to use hydrogels prepared with 20 mg/ml TA for all subsequent in vitro and in vivo studies.

### TA release in response to flare-associated enzymes

**Fig. 2:**
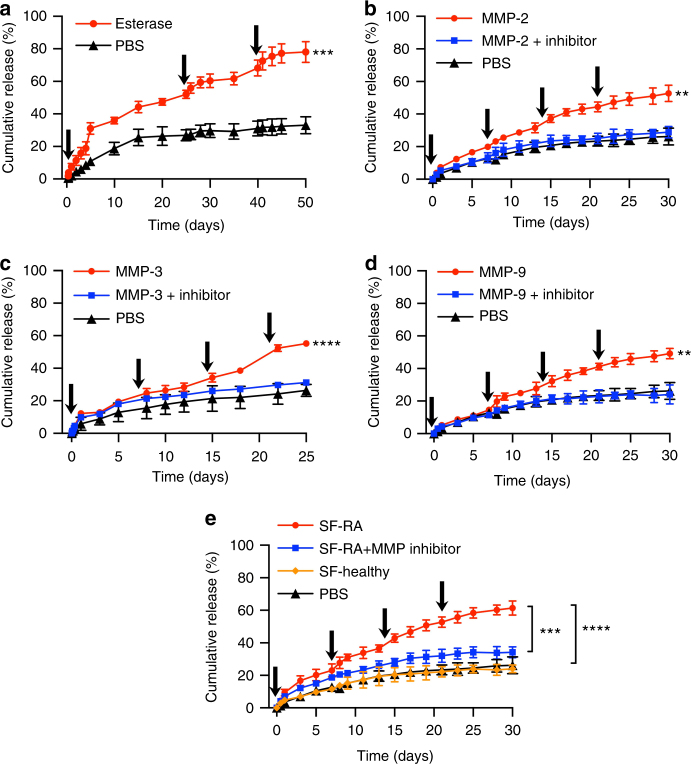
TG-18 hydrogel has long-term hydrolytic and encapsulation stability in PBS and exhibits on-demand release of encapsulated TA. **a** In vitro release kinetics of TA from TG-18 hydrogel in PBS at 37 °C without or with esterase (*T. lanuginosus* lipase, 200 U/ml). Fresh enzyme was added at the indicated time points (arrows). ***P < 0.001 compared with PBS on day 50. **b**–**d** In vitro release kinetics of TA from TG-18 hydrogel in PBS at 37 °C without or with MMP-2 (1.5 µg/ml), MMP-3 (5 µg/ml), or MMP-9 (1 µg/ml). Fresh enzyme or enzyme+MMP inhibitor was added at the indicated time points (arrows). **P < 0.01 compared with PBS or MMP-2 (MMP-9)+inhibitor on day 30 and ****P < 0.0001 compared with PBS or MMP-3+inhibitor on day 25. **e** In vitro release kinetics of TA in response to synovial fluid from human rheumatoid arthritis joints (SF-RA) and synovial fluid from healthy human joints (SF-healthy). Two hundred microliters of fresh synovial fluid or synovial fluid + MMP inhibitor cocktail were added at the indicated time points (arrows). ****P < 0.0001 and ***P < 0.001 on day 30. *P*-values were determined by Student’s *t*-test with Welch’s correction in **a** and one-way Anova with Tukey’s post hoc analysis in **b**–**e**. Data are means ± SD of technical repeats (*n* = 3, experiments performed at least twice)

Acute flares of IA are associated with upregulated expression of matrix metalloproteinases (MMPs) and other tissue-degrading enzymes^12,16^. Therefore, we investigated the ability of TG-18 hydrogel to disassemble and release the encapsulated TA in response to defined enzymatic activities in vitro. Drug-loaded hydrogels were incubated in PBS (pH 7.4) at 37 °C without or with matrix metalloproteinase-2 (MMP-2) (1.5 µg/ml), matrix metalloproteinase-3 (MMP-3) (5 µg/ml), or MMP-9 (1 µg/ml). Enzyme concentrations were chosen based on the values reported for synovial fluid from RA patients^12^. As control, we incubated TA-loaded TG-18 hydrogel with an esterase, *T. lanuginosus* lipase (200 U/ml). To mimic periodic IA flares, fresh enzyme was added to the release medium (PBS) at multiple time points. In the absence of enzyme, TA-loaded TG-18 hydrogel demonstrated excellent stability to non-specific hydrolysis in PBS, with less than 25% cumulative release of TA over a period of 50 days and no substantial burst release (Fig. 2a). Repeated addition of esterase or MMPs increased the cumulative drug release (Fig. 2a–d), which was suppressed when an MMP inhibitor cocktail was added together with the MMPs (Fig. 2b–d). Repeated pulses of enzyme resulted in significantly higher cumulative drug release compared with a single pulse (Supplementary Fig. 3) and the amount of cumulatively released TA correlated with the dose of the enzyme added into the release medium (Supplementary Fig. 4).

### TA release in response to synovial fluid from RA joints

We then investigated the release of TA from TG-18 hydrogel under conditions that more closely resemble the inflammatory milieu during IA flare. Drug-loaded hydrogel was incubated in PBS without or with the addition of synovial fluid from human RA joints or synovial fluid from healthy human joints. Similar to the studies with defined enzyme activities, synovial fluid was added at multiple time points during the experiment. Strikingly, synovial fluid from human RA joints but not from healthy human joints significantly increased the cumulative TA release compared to PBS (Fig. 2e). Addition of the MMP inhibitor cocktail suppressed TA release triggered by RA synovial fluid (Fig. 2e), confirming that the increase in drug release observed in the presence of arthritic synovial fluid was due to enzyme-mediated hydrogel disassembly.

### In vitro biocompatibility of TA-loaded hydrogel

**Fig. 3:**
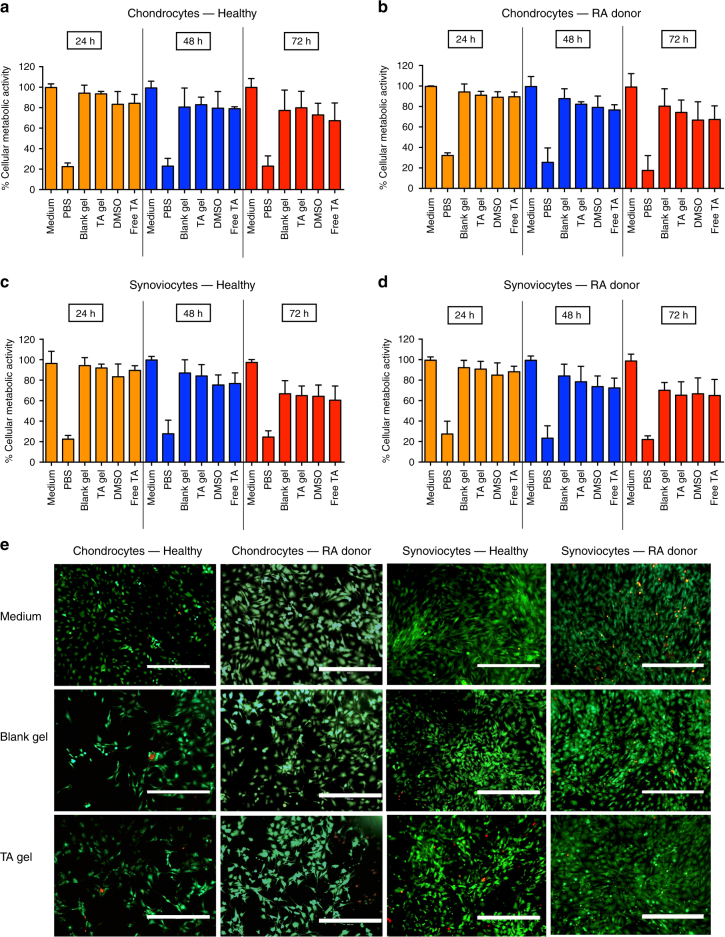
TA-loaded hydrogel is biocompatible with cells from healthy and arthritic human joints. **a**–**d** Primary human chondrocytes or synoviocytes from healthy donors or RA patients were incubated in a 96-well transwell plate in medium, PBS, or in medium with 50 µl blank hydrogel (Blank gel), 50 µl TA-loaded TG-18 hydrogel (TA gel), DMSO (equivalent to 50 µl hydrogel) or free drug (Free TA, equivalent to 50 µl TA-loaded TG-18 hydrogel) added to the upper chamber of the transwell. After 24, 48, and 72 h of incubation, metabolic activity was determined by XTT assay. **e** Representative fluorescence microscopy images after live/dead staining of primary human chondrocytes or synoviocytes from healthy donors or RA patients incubated for 72 h in medium or medium with blank hydrogel (Blank gel) or TA-loaded TG-18 hydrogel (TA gel) added to the upper chamber of the transwell (Scale bar: 400 µm). Viable cells stain green with calcein-AM, whereas dead cells stain red with ethidium homodimer-1. Data in **a**–**d** are means ± SD of technical repeats (*n* = 3, experiment performed at least twice)

Next, we investigated the in vitro biocompatibility of TG-18 hydrogel with primary human chondrocytes and synoviocytes isolated from healthy donors and RA patients. Cells were incubated in a 96-well transwell plate in medium, PBS, or in medium with blank hydrogel, TA-loaded TG-18 hydrogel, DMSO (equivalent to hydrogel), or free TA (equivalent to TA-loaded TG-18 hydrogel) added to the upper chamber. Metabolic activity was determined by 2,3-bis-(2-methoxy-4-nitro-5-sulfophenyl)-2*H*-tetrazolium-5-carboxanilide (XTT) assay after 24, 48, and 72 h of incubation. Cells incubated in PBS showed a significant reduction in metabolic activity compared to cells incubated in medium. In contrast, chondrocytes and synoviocytes incubated in the presence of blank or TA-loaded TG-18 hydrogel demonstrated greater than 65–70% metabolic activity at all time points (Fig. 3a–d), which is consistent with biocompatibility of these hydrogel formulations according to published reports for assessing in vitro toxicity of biomaterials^17^. Live/dead staining with calcein-AM/ethidium homodimer-1 also did not show significant loss of cell viability or changes in cell morphology in chondrocytes or synoviocytes incubated with blank or TA-loaded hydrogel (Fig. 3e). On the other hand, control cells treated with 70% methanol solution for 30 min showed significant cell death (Supplementary Fig. 5).

### Anti-inflammatory response of TA-loaded hydrogel in vitro

We also confirmed that the encapsulation of TA in TG-18 hydrogel does not compromise the activity of TA. Human macrophages, differentiated from THP-1 monocytes, were stimulated with lipopolysaccharide (LPS) in a transwell plate in the presence of blank hydrogel, TA-loaded TG-18 hydrogel, DMSO (equivalent to hydrogel), or free TA (equivalent to TA-loaded TG-18 hydrogel) added to the upper chamber. After 24 h of incubation, supernatant was assayed for tumor necrosis factor (TNF)-α and interleukin-10 (IL-10) by ELISA. Free TA and TA-loaded TG-18 hydrogel resulted in similar reductions in TNF-α secretion, while increasing IL-10 secretion (Supplementary Fig. 6), demonstrating that the anti-inflammatory effect of TA in the hydrogel formulation is not compromised.

### TG-18 hydrogel disassembly in vivo

Having shown that TG-18 hydrogel disassembles and releases the encapsulated agent on-demand in vitro, we investigated hydrogel disassembly in vivo using the murine K/BxN serum-transfer model of IA^13,14^. In this model, serum from arthritic K/BxN mice is injected intraperitoneally (i.p.) into naive C57BL/6J mice on days 0 and 2 resulting in a highly predictable monophasic arthritis flare in the forepaws and feet. Moreover, arthritis severity can be titrated by adjusting the injected volume of K/BxN serum.

**Fig. 4:**
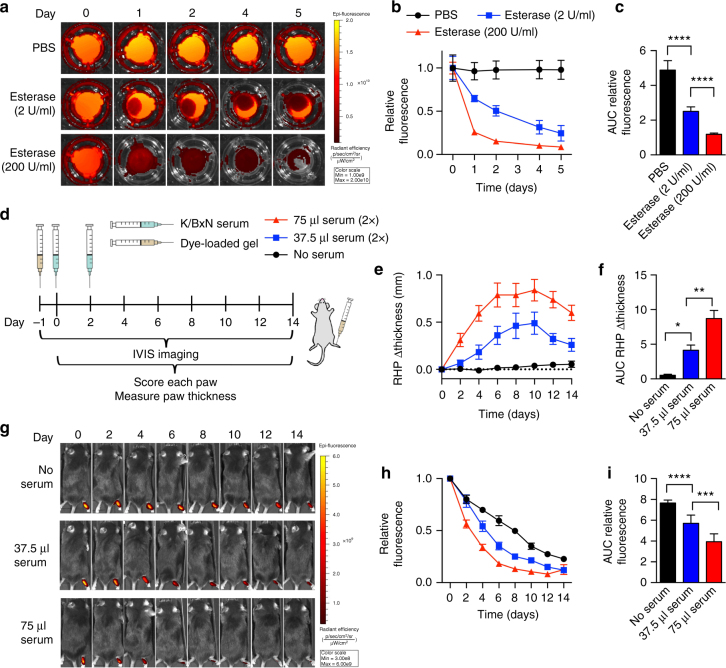
TG-18 hydrogel disassembly correlates with arthritis severity. **a** DiR-loaded hydrogels were incubated in PBS without or with esterase (*T. lanuginosus* lipase, 2 or 200 U/ml). To quantify fluorescence signals at each time point, transwell inserts with hydrogel were temporarily removed from the plate and placed on a new plate for imaging using an in vivo imaging system (IVIS). Images of a representative well from each experimental group are shown. **b**, **c** Relative fluorescence curves (normalized to day 0) for hydrogels inclubated without or with esterase and their area under the curves (AUCs) (****P < 0.0001). **d** Experimental outline: Mice were injected with fluorescent dye-loaded hydrogel (4 µl) into the right hindpaw (RHP) on day −1. Arthritis was subsequently induced with two i.p. injections of 37.5 or 75 μl KBx/N serum on day 0 and day 2. Control animals received no serum. Every other day, animals were imaged using IVIS, arthritis severity was scored clinically, and paw swelling was measured with calipers. **e**, **f** Change in RHP thickness curves and their AUCs (*P < 0.05 and **P < 0.01). **g** IVIS images of a representative animal from each experimental group. **h**, **i** Relative fluorescence (normalized to day 0) measured over the RHP and AUCs (***P < 0.001 and ****P < 0.0001). Data in **b**, **c** are means ± SD of technical repeats (*n* = 6, experiment performed twice). *P*-values were determined using one-way Anova with Tukey’s post hoc analysis. Data in **b**, **c** are means ± SD of technical repeats (*n* = 6, experiment performed at least twice). Data in **e**, **f** and **h**, **i** are means ± SEM (*n* = 6 mice/group, experiment performed twice)

To monitor hydrogel disassembly, TG-18 hydrogel was loaded with the fluorescent dye 1,1′-dioctadecyl-3,3,3′,3′-tetramethylindotricarbocyanine iodide (DiR). DiR-loaded TG-18 hydrogel was stable in PBS at 37 °C in vitro, whereas exposure to *T. lanuginosus* lipase (2 or 200 U/ml) resulted in dose-dependent loss of fluorescence (Fig. 4a–c). Arthritis-induced hydrogel disassembly could thus be quantified by in vivo imaging as loss of fluorescence over time.

To investigate flare-responsive hydrogel disassembly in vivo, we injected mice with 4 μl of DiR-loaded hydrogel into the right hindpaw on day −1. Arthritis was subsequently induced by i.p. injections on day 0 and day 2 of either 37.5 or 75 μl K/BxN serum. Control animals received no serum. We imaged the animals every other day using an in vivo imaging system (IVIS). Arthritis severity was scored clinically based on a previously described scoring system^18,19^ and paw swelling was measured with calipers (Fig. 4d). As expected, mice who received 2 × 75 μl of K/BxN serum had more severe arthritis than mice receiving 2 × 37.5 µl, documented by the change in right hindpaw thickness (Fig. 4e, f), right hindpaw clinical scores, total change in paw thickness, and total clinical scores (Supplementary Fig. 7). Fluorescence signal was detectable over the right hindpaw in arthritic mice and in healthy control animals for the entire 2 weeks of the study (Fig. 4g, h). Importantly, the fluorescence signal decayed more rapidly in the arthritic animals. Moreover, animals injected with 2 × 75 µl serum showed a significantly more rapid decay of fluorescence than animals injected with 2 × 37.5 µl serum who had less severe arthritis (Fig. 4g–i). These results demonstrate that TG-18 hydrogel is responsive to arthritis flare activity in vivo resulting in on-demand disassembly and release of the encapsulated agent controlled by the intensity of the inflammatory stimulus.

### Efficacy of TA-loaded hydrogel in vivo

**Fig. 5:**
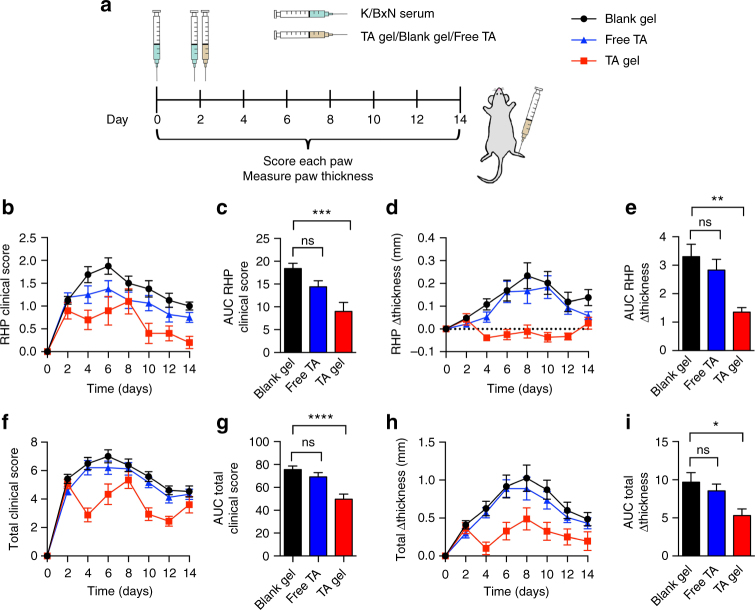
Local delivery of TA encapsulated in TG-18 hydrogel improves therapeutic efficacy compared to free TA in a mouse model of IA. **a** Experimental outline: Arthritis was induced by i.p. injection of 37.5 µl K/BxN serum on day 0 and day 2. Immediately after the second dose of K/BxN serum, TA-loaded TG-18 hydrogel (TA gel, 20 mg TA/ml, 4 µl), free TA (20 mg TA/ml, 4 µl), or blank TG-18 hydrogel (Blank gel, 4 µl) were injected into the right hindpaw. Every other day, arthritis severity was scored clinically and paw swelling was measured with calipers. **b**, **c** RHP clinical score curves and their AUCs (***P < 0.001). **d**, **e** Change in RHP thickness curves and their AUCs (**P < 0.01). **f**, **g** Total clinical score curves and their AUCs (****P < 0.0001). **h**, **i** Change in total paw thickness curves and their AUCs (*P < 0.05). *P-*values were determined by one-way Anova with Tukey’s post hoc analysis. Data are means ± SEM (*n* = 18–20 mice per group, data were pooled from three independent experiments)

We then investigated the therapeutic efficacy of TA-loaded TG-18 hydrogel. To this end, arthritis was induced by i.p. injections of K/BxN serum (37.5 ul) on day 0 and day 2. Immediately after the second dose of K/BxN serum, TA-loaded TG-18 hydrogel, free TA (equivalent to TA-loaded TG-18 hydrogel), or blank TG-18 hydrogel was injected into the right hindpaw (Fig. 5a). Treatment with TA-loaded TG-18 hydrogel reduced arthritis severity in the injected right hindpaw compared to animals treated with blank hydrogel (vehicle control) (Fig. 5b–e). Free TA did not have a significant therapeutic effect in this experiment, likely due to rapid clearance from the injection site as reported previously^20,21^. We also observed a reduction of change in total paw thickness and total clinical score in animals treated with TA-loaded hydrogel compared with the other groups (Fig. 5f–i). This was largely, although not entirely, explained by the almost complete abrogation of inflammation in the right hindpaw injected with TA-loaded hydrogel.

### Efficacy of TA-loaded hydrogel in vivo in severe arthritis

**Fig. 6:**
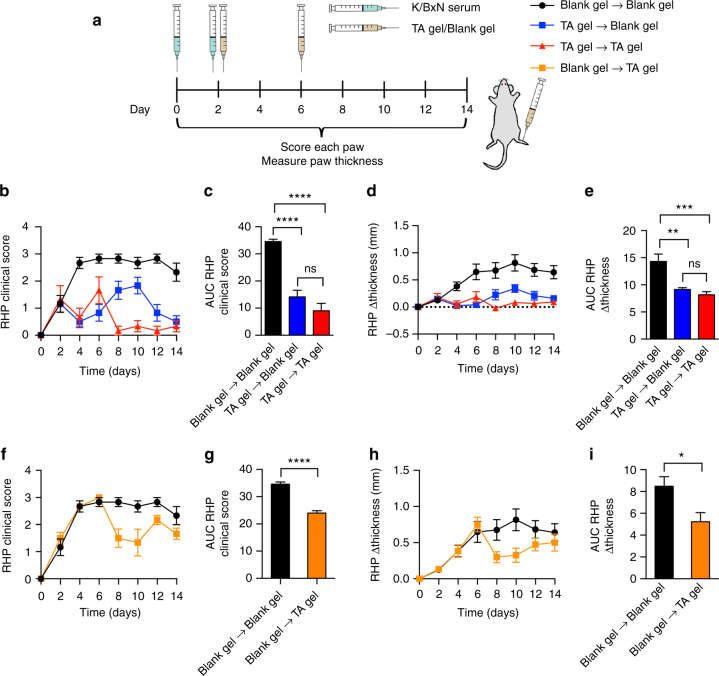
Local delivery of TA-loaded TG-18 hydrogel demonstrates therapeutic efficacy in severe arthritis. **a** Experimental outline: Arthritis was induced by i.p. injection of 75 µl K/BxN serum on day 0 and day 2. Immediately after the second dose of K/BxN serum, animals were randomized into four groups. The right hindpaw of each mouse was injected with hydogel on day 2 and day 6. The (Blank gel→Blank gel) group received blank hydrogel (4 µl) twice, (TA gel→Blank gel) mice received TA-loaded TG-18 hydrogel (20 mg TA/ml, 4 µl) on day 2 and blank hydrogel on day 6, the (TA gel→TA gel) group received two doses of TA-loaded TG-18 hydrogel, and the (Blank gel→TA gel) group received blank hydrogel on day 2 and TA-loaded TG-18 hydrogel on day 6. Every other day, arthritis severity was scored clinically and paw swelling was measured with calipers. **b**, **c** RHP clinical score curves and their AUCs and **d**, **e** Change in RHP thickness curves and their AUCs for (Blank gel → Blank gel), (TA gel → Blank gel), and (TA gel → TA gel) (**P < 0.01, ***P < 0.001, ****P < 0.0001). **f**, **g** RHP clinical score curves and their AUCs and **h**, **i** Change in RHP paw thickness curves and their AUCs for (Blank gel → Blank gel) and (Blank gel → TA gel) (*P < 0.05, ****P < 0.0001). For **c**, **e**, *P*-values were determined by one-way Anova with Tukey’s post hoc analysis and for **g**, **i**, by Student’s *t-*test with Welch’s correction. Data are means ± SEM (*n* = 6 mice per group)

Locally administered TA-loaded TG-18 hydrogel was also effective when the volume of arthritis-inducing K/BxN serum was doubled to 75 µl in order to induce more severe disease (Fig. 6). In this experiment, mice received two consecutive injections of either blank or TA-loaded TG-18 hydrogel into the right hindpaw on day 2 and day 6 (Fig. 6a). Treatment with a single dose of TA-loaded TG-18 hydrogel on day 2 (TA gel→Blank gel) reduced arthritis severity in the right hindpaw compared to animals treated with blank hydrogel on days 2 and 6 (Blank gel→Blank gel) (Fig. 6b–e). The effect of a single dose of TA-loaded TG-18 hydrogel injected on day 2 (TA gel→Blank gel) on clinical score and paw swelling was statistically not different from two doses of TA-loaded TG-18 hydrogel, injected on days 2 and 6 (TA gel→TA gel) (Fig. 6b–e), although suppression of disease appeared more complete in mice receiving two injections of drug-loaded hydrogel. Injecting a single dose of TA-loaded hydrogel at the peak of disease severity, i.e. day 6 (Blank gel→TA gel) was also efficacious at reducing arthritis severity post injection (Fig. 6f–i). We also observed a reduction of change in total paw thickness and total clinical score in animals treated with TA-loaded TG-18 hydrogel (Supplementary Fig. 8). This was largely, although not entirely, explained by the almost complete abrogation of inflammation in the right hindpaw injected with TA-loaded hydrogel. Taken together, our data document the promise of TG-18 hydrogel as a drug delivery system in IA to improve the therapeutic efficacy of locally delivered drugs via arthritis flare-responsive drug release.

## Discussion

Several intra-articular drug delivery approaches have been explored to increase the joint residence time of locally administered therapeutics^3–11^. So far, these strategies have shown only modest benefits over current clinical formulations when tested in clinical trials^2^. For instance, a recent phase 2 study evaluated TA encapsulated into biodegradable poly(lactic-co-glycolic) acid microparticles (FX006, Flexion Therapeutics) in knee osteoarthritis^22,23^. Compared to standard treatment with intra-articular bolus injection of TA (Kenalog-40), a single injection of the FX006 formulation resulted in statistically significant but clinically minimal improvement in pain relief with similar duration of the therapeutic effect (~12 weeks)^22–24^.

Most of the previously described systems provide sustained drug release over time. We reasoned that an autonomous drug delivery system, which titrates drug release to the current disease activity in the joint, might improve therapeutic outcomes. The concept guiding the development of TG-18 hydrogel has been to generate an intra-articular drug reservoir which releases drug in response to enzymes overexpressed in inflamed joints. Prior studies of inflammation-responsive drug delivery systems in IA relied on polymeric particles releasing drug cargo in response to the low pH in inflamed synovial fluid^25–27^. These drug-loaded particles were administered systemically and entered inflamed joints from the blood stream. However, the therapeutic benefits from each injection lasted only 48–72 h in preclinical models^26^. Moreover, intravenously injected nano- and microparticles tend to accumulate in the liver and spleen via the reticuloendothelial system^26^, increasing the risk for toxic side effects. Finally, the synthesis of pH-responsive polymer conjugates like hyaluronic acid-5*β*-cholanic acid conjugate based nanoparticles^27^ and *N*-(2-hydroxypropyl)methacrylamide copolymer-dexamethasone conjugate^28,29^ is complex, which limits scalability and translatability. TG-18 on the other hand, can be coaxed to form self-assembling hydrogel using a simple process. There is no chemical modification required for this platform, which makes our approach amenable to large-scale manufacturing and can enable rapid translation.

The enzyme-cleavable linker in TG-18 is an ester bond. However, enzymes other than bona fide esterases can cleave ester bonds. This includes MMPs, which are overexpressed during IA flares^12,30–32^. The mechanism involves binding of the Zn (II) ion of MMPs to external water molecules, followed by their deprotonation. This results in the formation of Zn(II)-bound hydroxides, which act as nucleophiles that attack electrophilic substrates including carboxy esters like those found in TG-18^33,34^. Previous attempts to develop MMP-responsive drug delivery systems involved the synthesis of specific MMP-cleavable peptides such as GGRMSMPV^35,36^, which were then incorporated into the drug delivery system by chemical conjugation. In contrast, our approach is peptide-free and utilizes off-the shelf materials which do not require chemical modification and is amenable to large-scale manufacturing. Despite the simplicity of the formulation, we did not observe substantial burst release of TA from TG-18 in vitro. In fact, spontaneous cumulative drug release was only 20% over 10 days in vitro. In contrast, amphiphillic polyphosphazene-based polymeric micelles loaded with indomethacin showed 95% cumulative drug release within 5 h^6.^ Burst release of drug is undesirable as it results in rapid exhaustion of the drug depot and systemic absorption of the released therapeutic can lead to systemic toxicity.

A single pulse of RA synovial fluid triggered the enzymatic release of TA from TG-18 hydrogel, but did not result in 100% cumulative release. The bulk of TG-18 hydrogel comprises multiple self-assembled interdigitated bilayers, arranged as concentric layers in each fiber. Presumably, only TG-18 available on the fiber surface is accessible to enzyme-mediated cleavage of the ester bond, ensuring that the hydrogel is not completely unstable in the inflamed joints.

TG-18 hydrogel is a versatile platform that can encapsulate a wide range of therapeutics and is not limited to a single drug. We have previously shown the utility of TG-18 for local delivery of tacrolimus in a preclinical model of vascularized composite allo-transplantation^15^. We have also identified multiple other GRAS amphiphiles, including ascorbyl palmitate^37^, that can self-assemble to form inflammation-responsive hydrogels. These ampiphiles can be leveraged in the future to develop formulations with maximum loading and stability of the therapeutics of interest, enabling future expansion of the platform.

Our study has certain limitations. First, while our goal has been to develop a system that releases the drug on-demand exclusively in response to inflammation with minimal release under non-inflammatory conditions, we did observe baseline drug and dye release under non-inflammatory conditions in vitro and in vivo. This may be due to non-enzymatic hydrolysis of TG-18 in aqueous environments, resulting in gel disassembly or due to passive diffusion of non-encapsulated or surface-absorbed drug or dye. Formulations of next-generation flare-responsive hydrogels could incorporate modifications that reduce or eliminate spontaneous gel disassembly and drug release. For example, the hydrogel composition maybe tweaked to reduce baseline non-enzymatic hydrolysis, for instance by combining TG-18 with other amphiphiles that are not responsive to inflammatory enzymes. Alternatively, the hydrogel preparation and drug encapsulation process maybe modified to minimize the amount of surface-absorbed drug or remove it prior to injection.

Secondly, one would like to administer drug-loaded hydrogel directly into joints in preclinical models to mimic the proposed use in humans and determine true joint residence times. However, mouse joints are very small which imposes strict limits on the total injectable volume and, for all practical purposes, limits intra-articular injections to the knee joints. In K/BxN serum-transfer arthritis, which we choose for this study because of its excellent reproducibility and titratability, the knee joints are not affected^19,38^. Using animal models with larger joints (i.e. rat, rabbit, goat, pig) would permit the injection of volumes similar to what one would administer in humans. More extensive in vivo biocompatibility evaluation will also be required in large animal models prior to clinical trials in humans. Future in vivo studies comparing flare-responsive drug delivery with sustained release will be important to establish the therapeutic advantage of arthritis flare-responsiveness drug delivery approach. Validating this platform in other mouse arthritis models will also be important for its clinical translation. Finally, future studies will explore the delivery of other established or investigational drugs, followed by in vivo mechanistic studies involving detailed histological analysis and measurements of relevant cytokines and other inflammatory mediators in the joint.

In conclusion, we have developed an arthritis flare-responsive drug delivery platform for on-demand intra-articular release of therapeutics. This system has the potential to provide the optimal amount of a therapeutic at a time when it is needed, thereby maximizing therapeutic efficacy and prolonging the duration of the therapeutic benefit.

## Methods

### Preparation of hydrogel and drug encapsulation

To prepare blank hydrogel (10% w/v), 100 mg of triglycerol monostearate (TG-18) (AK Scientific) was weighed into a glass scintillation vial, followed by the addition of 1 ml DMSO–water mixture (1:4 volume ratio). The mixture was heated to 60–80 °C until TG-18 dissolved. The vial was then placed on a flat surface and allowed to cool to room temperature for 15–30 min, resulting in hydrogel formation. Gelation was complete when no gravitational flow was observed upon inversion of the vial. TA (Sigma Aldrich) or 1,1′-dioctadecyl-3,3,3′,3′-tetramethylindotricarbocyanine iodide (DiR) (Thermo Fisher Scientific) were added to the vial together with TG-18, for a final concentration of 10–40 mg/ml TA and 100 µg/ml DiR. For in vivo studies, we transferred the heated hydrogel preparations to glass syringes prior to gelation. Hydrogel morphology was characterized by HR-SEM (Zeiss Merlin High-resolution SEM, acceleration voltage, 2 kV). For electron microscopic analysis, xerogels were prepared by lyophilizing the gels. Small amounts of xerogels were placed on carbon tape attached to aluminum grids and coated with a thin layer of gold using a sputtering machine.

### Cell culture

Primary human synoviocytes and chondrocytes from healthy and RA donors (Articular Engineering) were cultured in T-75 flasks (VWR) at 37 °C and 5% CO_2_ in synoviocyte growth medium or chondrocyte growth medium (Articular Engineering), respectively. Media were supplemented with 10% fetal calf serum (Articular Engineering) and 1% penicillin–streptomycin (Thermo Fisher Scientific).

Human THP-1 monocytes (American Type Culture Collection) were cultured in T-75 flasks at 37 °C and 5% CO_2_ in RPMI-1640 (American Type Culture Collection) supplemented with 10% fetal bovine serum (Atlanta Biologicals), 0.05 M β-mercaptoethanol (EMD Millipore), and 1% penicillin–streptomycin. For differentiation into macrophages, 1×10^6^ cells were treated with 50 nM phorbol 12-myristate 13-acetate (Sigma Aldrich) for 3 days. Cell lines were authenticated by their sources using morphology, karyotyping, and PCR based approaches.

### Enzyme-responsive release

Enzyme-responsive release of TA from TG-18 hydrogel was performed at pH 7.4 and 37 °C. TA-loaded hydrogels (50 μl, 20 mg TA/ml) were placed in dialysis tubing (8–10 kDa molecular weight cut-off; Spectrum Labs) and suspended in PBS (950 μl) without or with one of the following enzymes: esterase (*T. lanuginosus* lipase, 200, 400, or 800 U/ml) (Sigma Aldrich); recombinant human MMP-2 (1.5 μg/ml) (Sigma Aldrich); recombinant human MMP-3 (5 µg/ml) (Sigma Aldrich), and recombinant human MMP-9 (1 μg/ml) (Sigma Aldrich). In some experiments, MMP-2/9 Inhibitor II (Sigma Aldrich) or MMP-3 Inhibitor II (Sigma Aldrich) was added along with the MMPs. Fresh enzyme or enzyme + MMP inhibitor were added at multiple time points as indicated in the figure legends. The dialysis bags filled with hydrogel in release medium were placed in 45 ml sink medium (PBS), and incubated at 37 °C with a shaking speed of 150 rpm. At each time point, an aliquot (1 ml) of sink medium was removed and replenished with the same volume of fresh PBS to ensure constant sink conditions. Aliquots were lyophilized and dissolved in 250 μl methanol, followed by high-performance liquid chromatography (HPLC) (Agilent 1100 quaternary LC pump liquid chromatograph, Zorbax SB C-18 column, 250 × 4.6 mm, 5 µm).

### In vitro release in response to human synovial fluid

TA-loaded TG-18 hydrogels (50 μl, 20 mg TA/ml) were placed in dialysis tubing (8–10 kDa molecular weight cut-off) and suspended in PBS (650 μl). The dialysis bags were placed in 45 ml sink medium (PBS), and incubated at 37 °C with a shaking speed of 150 rpm. To evaluate drug release in response to synovial fluid from healthy and human RA joints, 200 µl synovial fluid from human RA joints or healthy human joints (Articular Engineering) or an equal volume of PBS was added to the dialysis bag at multiple time points. In some experiments, MMP-2/9 Inhibitor II was added together with synovial fluid from human RA joints. Aliquots taken from the sink medium (PBS) were analyzed using HPLC, as described above.

### In vitro biocompatibility

Cultured primary human synoviocytes and chondrocytes were harvested at 80% confluency and seeded (seeding density: 20,000 cells/well) in 96-well plates (Corning) containing transwell inserts (Thermo Fisher Scientific). After 24 h of culture, medium was replaced with fresh medium, PBS, or fresh medium with 50 µl blank hydrogel, 50 µl TA-loaded hydrogel, DMSO (equivalent to 50 µl gel), or free TA (equivalent to 50 µl TA-loaded hydrogel) added to the upper chamber. After 24, 48, and 72 h of incubation, cellular metabolic activity was determined using 2,3-bis-(2-methoxy-4-nitro-5-sulfophenyl)-2*H*-tetrazolium-5-carboxanilide (XTT) cell proliferation assay kit (American Type Culture Collection) and was normalized relative to cells treated with only medium to determine percentage metabolic activity.

Alternatively, primary human synoviocytes and chondrocytes were seeded on coverslips placed in 24-well plates (Corning) containing transwell inserts (Fisher Scientific) at 50,000–100,000 cells/well. After 24 h of culture, medium was replaced with fresh medium or fresh medium with 75 µl blank hydrogel or 75 µl TA-loaded hydrogel added to the upper chamber. After 72 h of incubation, live-dead staining was performed using calcein-AM/ethidium homodimer-1 dye solutions from the LIVE/DEAD™ Viability/Cytotoxicity Kit as per the manufacturer’s instructions. Cells treated with 30% methanol for 30 min were used as positive control for cell death. Cells were imaged using EVOS FL Cell Imaging System (Thermo Fisher Scientific).

### In vitro anti-inflammatory activity

Human macrophages, differentiated from THP-1 cells in 24-well transwell plates, were incubated in medium or in medium with 100 µl blank hydrogel, 100 µl TA-loaded hydrogel, DMSO (equivalent to 100 µl hydrogel), or free TA (equivalent to 100 µl of TA-loaded hydrogel) added to the upper chamber of the transwell. LPS (100 ng/ml) was added in all wells to activate the macrophages. After 24 h of incubation, supernatants were assayed for TNF and IL-10 using commercial ELISA kits (BioLegend).

### Disassembly of DiR-loaded hydrogel in vitro

Aliquots of DiR-loaded hydrogel (100 µl) were added to the upper chambers of a PBS-filled 12-well transwell plate on day −2. The plate was then incubated in a 37 °C incubator shaker. On day 0, the PBS in each well was replaced with fresh PBS without or with *T. lanuginosus* lipase (2 or 200 U/ml). The plate was again incubated in a 37 °C incubator shaker and fluorescence signals were quantified on days 0, 1, 2, 4, and 5. To this end, the transwell inserts with the DiR-loaded hydrogels were temporarily removed, placed on a new plate, and imaged using an IVIS (IVIS Lumina II; PerkinElmer) at the Institute of Chemistry and Cell Biology–Longwood Screening Facility at Harvard Medical School.

### Mice

All in vivo experiments were performed in 6–10-week-old male C57BL/6J mice (Jackson Laboratory). The group sizes for each experimental design (at least six animals per treatment group) was determined based on the minimal number of animals needed to achieve a significant difference of *P* < 0.05 between experimental groups for the primary outcome of individual experiment using statistical analysis indicated in each figure legend. We randomly selected mice from the cage to assign them to different experimental groups. Experiments were performed in specific pathogen-free animal facilities at the Harvard T.H. Chan School of Public Health or Brigham and Women’s Hospital, or at the Institute of Chemistry and Cell Biology–Longwood Screening Facility at Harvard Medical School. Mice were housed under standard 12 h light–12 h dark conditions with ad libitum access to water and chow. All mouse studies were performed according to the institutional and National Institutes of Health (NIH) guidelines for humane animal use and in accordance with the Association for Assessment and Accreditation of Laboratory Animal Care. Protocols were approved by the Institutional Animal Care and Use Committee (IACUC) at Harvard Medical School.

### K/BxN serum-transfer model of IA

To induce IA, K/BxN serum (37.5 or 75 µl) was injected i.p. on experimental days 0 and 2. Every other day for 14 days, paw and ankle swelling were measured using calipers along with clinical indices as described^18,19,39^. Briefly, arthritis severity in each forepaw or ankle was rated from 0 (normal) to 3 (severe). Results are reported for the right hindpaw alone (RHP Δ thickness or RHP clinical score) or as the sum of all four paws (total Δ thickness or total clinical score).

### Hydrogel disassembly in vivo

Mice were injected with either DiR-loaded hydrogel (4 µl, 100 µg DiR/ml) or hydrogel co-loaded with DiR and TA (4 µl, 20 mg TA/ml) under the skin of the dorsal aspect of the right hindpaw on day −1. Arthritis was subsequently induced with two i.p. injections of 37.5 or 75 μl K/BxN serum on day 0 and day 2. Control animals received no serum. Every other day for 14 days, mice were anesthetized via isoflurane inhalation, and imaged using IVIS.

### In vivo therapeutic efficacy

Mice were injected with K/BxN serum (37.5 μl) i.p. on days 0 and 2. Immediately after the second dose of K/BxN serum, animals were randomized into three groups and injected with TA-loaded TG-18 hydrogel (4 µl, 20 mg TA/ml), free TA (4 µl, 20 mg TA/ml), or blank TG-18 hydrogel (4 µl) into the right hindpaw. Arthritis severity was measured every other day for 14 days^18,19,39^.

Alternatively, mice were injected with K/BxN serum (75 μl) i.p. on days 0 and 2. Immediately after the second dose of K/BxN serum, animals were randomized into four groups: Immediately after the second dose of K/BxN serum, animals were randomized into four groups: (Blank gel→Blank gel) group received blank hydrogel (4 µl) on days 0 and 2, (TA gel→Blank gel) group received TA-loaded hydrogel (4 µl, 20 mg TA/ml) on day 2 and blank hydrogel on day 6, (TA gel → TA gel) group received two doses of TA-loaded TG-18 hydrogel—one on day 2 and another on day 6, and (Blank gel → TA gel) group received blank hydrogel on day 2 and TA-loaded TG-18 hydrogel on day 6. Arthritis severity was measured every other day for 14 days^18,19,39^. The investigator performing the assessment of arthritis activity was unaware of group assignment of the individual mouse. Mice from different groups were co-housed.

### Statistics

Statistical analysis and graphing were done with Graphpad Prism. The two-tailed Student’s *t*-test with Welch’s correction (to stabilize the variance between different groups) was used to compare two experimental groups and one-way Anova with Tukey’s post hoc analysis was used for comparing more than two groups. To test for statistical significance of changes in paw thickness and clinical score, the area under curve for individual mice was calculated and group means were compared. The same approach was used to determine the statistical significance of differences in fluorescence signal decay in the in vitro or in vivo experiments with DiR-loaded hydrogel. For in vivo efficacy experiment performed in severe arthritis conditions, Grubb’s analysis (with alpha = 0.5) was performed to detect outliers, which resulted in the exclusion of one animal from the analysis. A value of *P* < 0.05 was considered statistically significant.

### Data availability

The data that support the findings of this study are available from the corresponding author upon request.

## Electronic supplementary material

Supplementary Information(PDF 2937 kb)
