# Author Correction: Towards an arthritis flare-responsive drug delivery system

**DOI:** 10.1038/s41467-018-04346-x

**Published:** 2018-05-11

**Authors:** Nitin Joshi, Jing Yan, Seth Levy, Sachin Bhagchandani, Kai V. Slaughter, Nicholas E. Sherman, Julian Amirault, Yufeng Wang, Logan Riegel, Xueyin He, Tan Shi Rui, Michael Valic, Praveen K. Vemula, Oscar R. Miranda, Oren Levy, Ellen M. Gravallese, Antonios O. Aliprantis, Joerg Ermann, Jeffrey M. Karp

**Affiliations:** 1 Center for Nanomedicine and Division of Engineering in Medicine, Department of Medicine, Brigham and Women’s Hospital, 02115 Boston, MA USA; 2 Harvard–Massachusetts Institute of Technology Division of Health Sciences and Technology, Massachusetts Institute of Technology, 02139 Cambridge, MA USA; 3 Harvard Medical School, 02115 Boston, MA USA; 4 Division of Rheumatology, Immunology and Allergy, Department of Medicine, Brigham and Women’s Hospital, 02115 Boston, MA USA; 5 Division of Rheumatology, Department of Medicine, University of Massachusetts Medical School, MA 01605 Worcester, USA; 6 Present Address: Institute for Stem Cell Biology and Regenerative Medicine (inStem), UAS-GKVK post, Bellary Road, 560065 Bangalore, India; 7 Present Address: Merck Research Laboratories, 33 Ave Louis Pasteur, 02115 Boston, MA USA

Correction to: *Nature Communications* 10.1038/s41467-018-03691-1, published online 03 April 2018.

In the original version of this Article, financial support was not fully acknowledged. The PDF and HTML versions of the Article have now been corrected to include support from the National Football League Players Association.

