# Supplementary material for: Towards an arthritis flare-responsive drug delivery system
Source: Nat Commun. 2018 Apr 3;9:1275. doi: 10.1038/s41467-018-03691-1 (PMC5882944; doi:10.1038/s41467-018-03691-1)
Supplement: Supplementary file 1 — Supplementary Information(PDF 2937 kb) [file 41467_2018_3691_MOESM1_ESM.pdf]

Towards an Arthritis Flare-Responsive Drug Delivery System

Nitin Joshi[1,2,3†], Jing Yan[3,4†], Seth Levy[3,4†], Sachin Bhagchandani[1], Kai V. Slaughter[1], Nicholas E. Sherman[1], Julian Amirault[1], Yu Feng Wang[1], Logan Riegel[1], Xueyin He[1], Tan Shi Rui[1], Michael Valic[1], Praveen K. Vemula[1,2,3,#], Oscar R. Miranda[1,2,3], Oren Levy[1,2,3], Ellen M. Gravallese[5], Antonios O. Aliprantis[3,4##], Joerg Ermann[3,4*], Jeffrey M. Karp[1,2,3*]

[1]Center for Nanomedicine and Division of Engineering in Medicine, Department of Medicine, Brigham and Women's Hospital, Boston, MA 02115, USA. [2]Harvard–Massachusetts Institute of Technology Division of Health Sciences and Technology, Cambridge, MA 02139, USA. [3]Harvard Medical School, Boston, MA 02115, USA. [4]Division of Rheumatology, Immunology and Allergy, Brigham and Women's Hospital, Boston, MA 02115, USA. [5]Department of Medicine, Division of Rheumatology, University of Massachusetts Medical School, Worcester, MA 01605. [#]Present affiliation: Institute for Stem Cell Biology and Regenerative Medicine (inStem), UAS-GKVK post, Bellary Road, Bangalore 560065, India. [##]Present affiliation: Merck Research Laboratories, 33 Ave Louis Pasteur Boston, MA 02115, USA. [†]These authors contributed equally to this work. [*]Correspondence and requests for materials should be addressed to J.M.K. (email: jmkarp@bwh.harvard.edu) and J.E. (email: jermann@bwh.harvard.edu).

Supplementary Figures

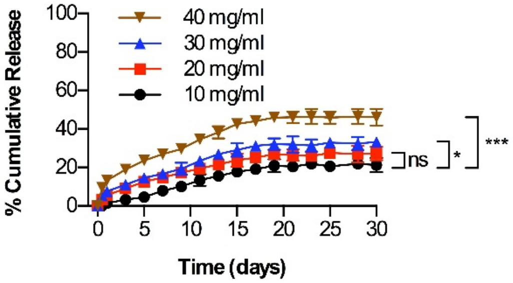

Supplementary Figure 1: TG-18 hydrogel shows dose-response relationship between TA concentration and its encapsulation stability. *In vitro* release kinetics of TA in PBS (pH 7.4) at 37°C comparing hydrogel formulations generated with same amount of TG-18 (10 % w/v), but loaded with different concentrations of TA. **P*<0.05 and ***P*<0.01 on day 30. *P* values were determined by one-way Anova with Tukey's post hoc analysis. Data are means ± SD for technical repeats (n = 3, experiment performed at least twice).

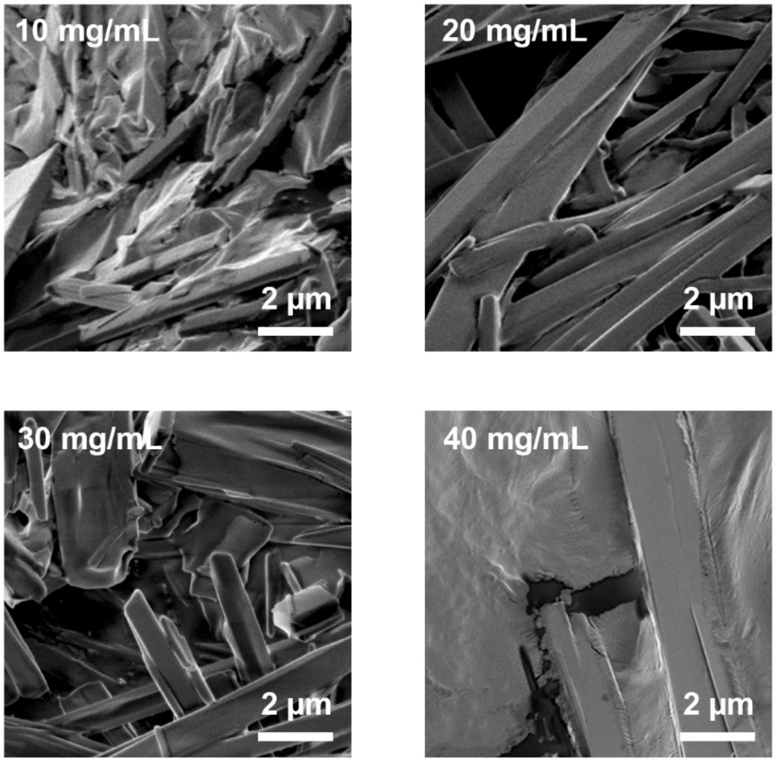

Supplementary Figure 2: High resolution scanning electron microscopy (HR-SEM) images of TA-loaded TG-18 hydrogels formulated with different TA concentrations. **(a)** 10 mg/ml; **(b)** 20 mg/ml; **(c)** 30 mg/ml and **(d)** 40 mg/ml.

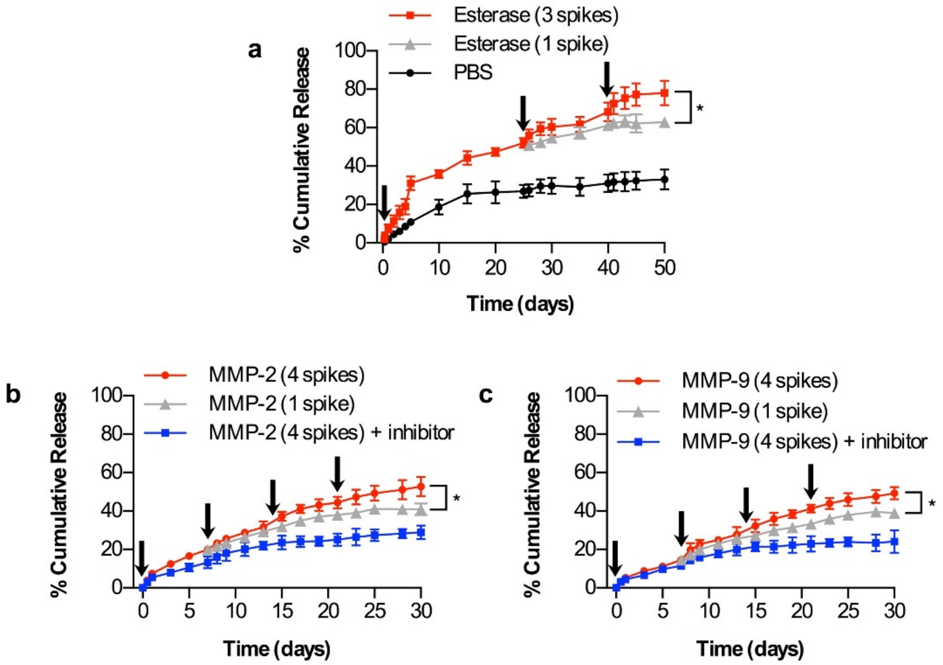

Supplementary Figure 3: A single pulse of enzyme results in less cumulative release of TA compared to multiple pulses. *In vitro* release kinetics of TA from TG-18 hydrogel in PBS without or with a single or multiple pulses of enzymes, including **(a)** esterase (200 U/ml), **(b)** MMP-2 (1.5 µg/ml) and **(c)** MMP-9 (1 µg/ml). Fresh enzyme or enzyme + inhibitor were added at the indicated time points (arrows). For single pulse based releases, enzyme was added only on day 0. For (a), **P*<0.05 on day 50 and for (b) and (c), **P*<0.05 on day 30. *P* values were determined by Students' *t* test. Data are means ± SD for technical repeats (n = 3, experiment performed at least twice).

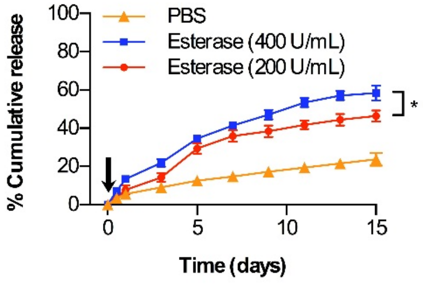

Supplementary Figure 4: TA-loaded TG-18 hydrogel shows dose-response relationship between enzyme concentration and TA release. *In vitro* release kinetics of TA from TG-18 hydrogels in PBS without or with different concentrations of esterase (T. *lanuginosus* lipase) (**P*<0.05). Enzyme was added at the indicated time

point (arrow). *P* values were determined by one-way Anova with Tukey's post hoc analysis. Data are means ± SD for technical repeats (n = 3, experiment performed at least twice).

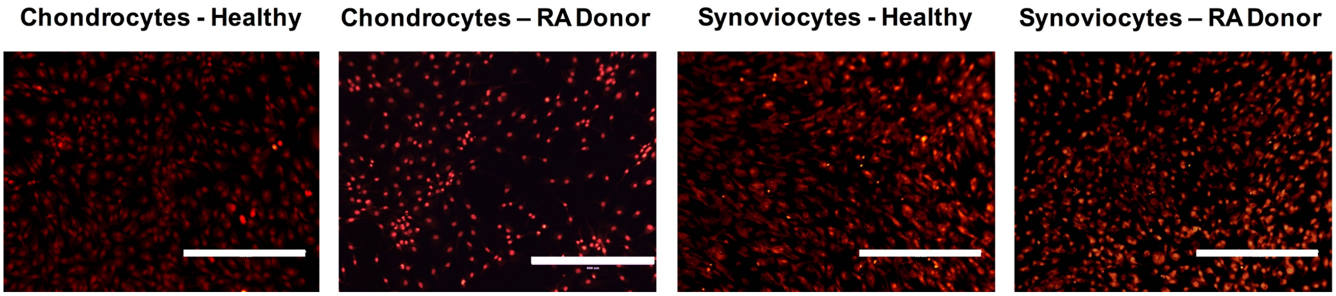

Supplementary Figure 5: Representative fluorescence microscopy images from the live/dead assay of control primary human chondrocytes or synoviocytes treated with 70% methanol solution for 30 minutes. Live/dead assay was performed using calcein-AM/ethidium homodimer-1 dye combination. Live cells stain green with the calcein-AM, whereas dead cells stain red with ethidium homodimer-1 (Scale bar: 400 μm).

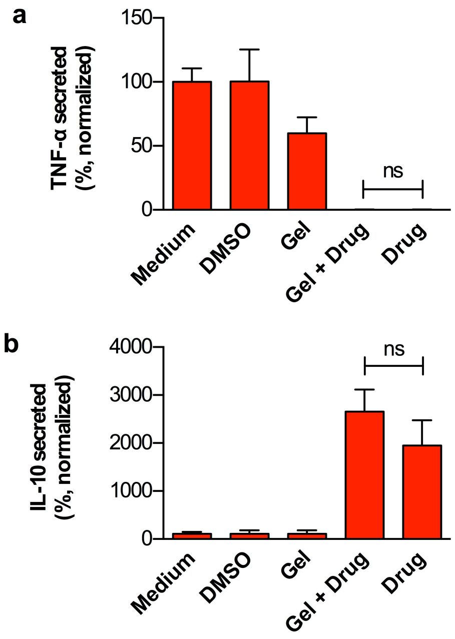

Supplementary Figure 6: TG-18 hydrogel does not compromise the therapeutic efficacy of TA *in vitro*. (a) TNF-α secretion and **(b)** IL-10 secretion from LPS stimulated human macrophages incubated in a transwell plate in medium (Medium) or in medium with 100 μl blank hydrogel (Gel), 100 μl TA-loaded hydrogel (Gel + Drug), DMSO (equivalent to 100 μl Gel) or free TA (Drug, equivalent to 100 μl Gel + Drug) added to the upper

chamber of the transwell. Following 24 h incubation, supernatant was assayed for TNF-α and IL-10 levels by ELISA, and normalized to the levels obtained for cells incubated with medium only. *P* values were determined by one-way Anova with Tukey's post hoc analysis. Data are means ± SD for technical repeats (n = 3, performed at least twice).

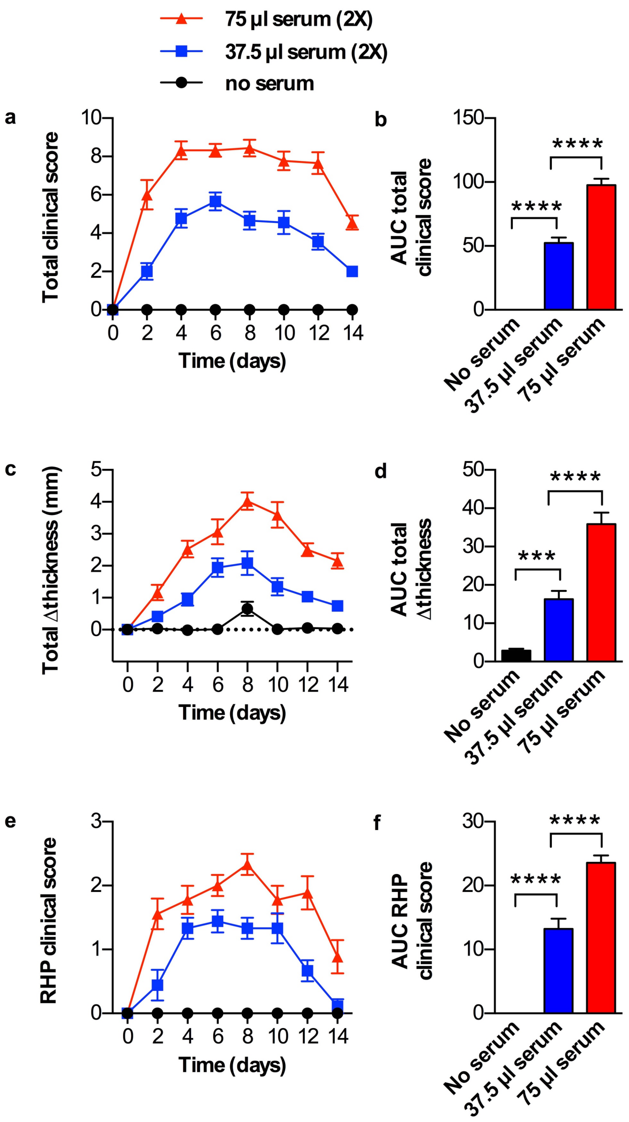

Supplementary Figure 7: Two doses of 75 µl K/BxN serum resulted in more severe arthritis than two doses of 37.5 µl serum. (a,b) Total clinical score curves and their AUCs (****P<0.0001). **(c,d)** Change in total paw thickness curves and their AUCs (***P<0.001 and ****P<0.0001). **(e,f)** RHP clinical score curves and their AUCs (****P<0.0001). P values were determined by one-way Anova with Tukey's post hoc analysis. Data are means ± SEM (n = 6 mice/group, experiment performed twice).

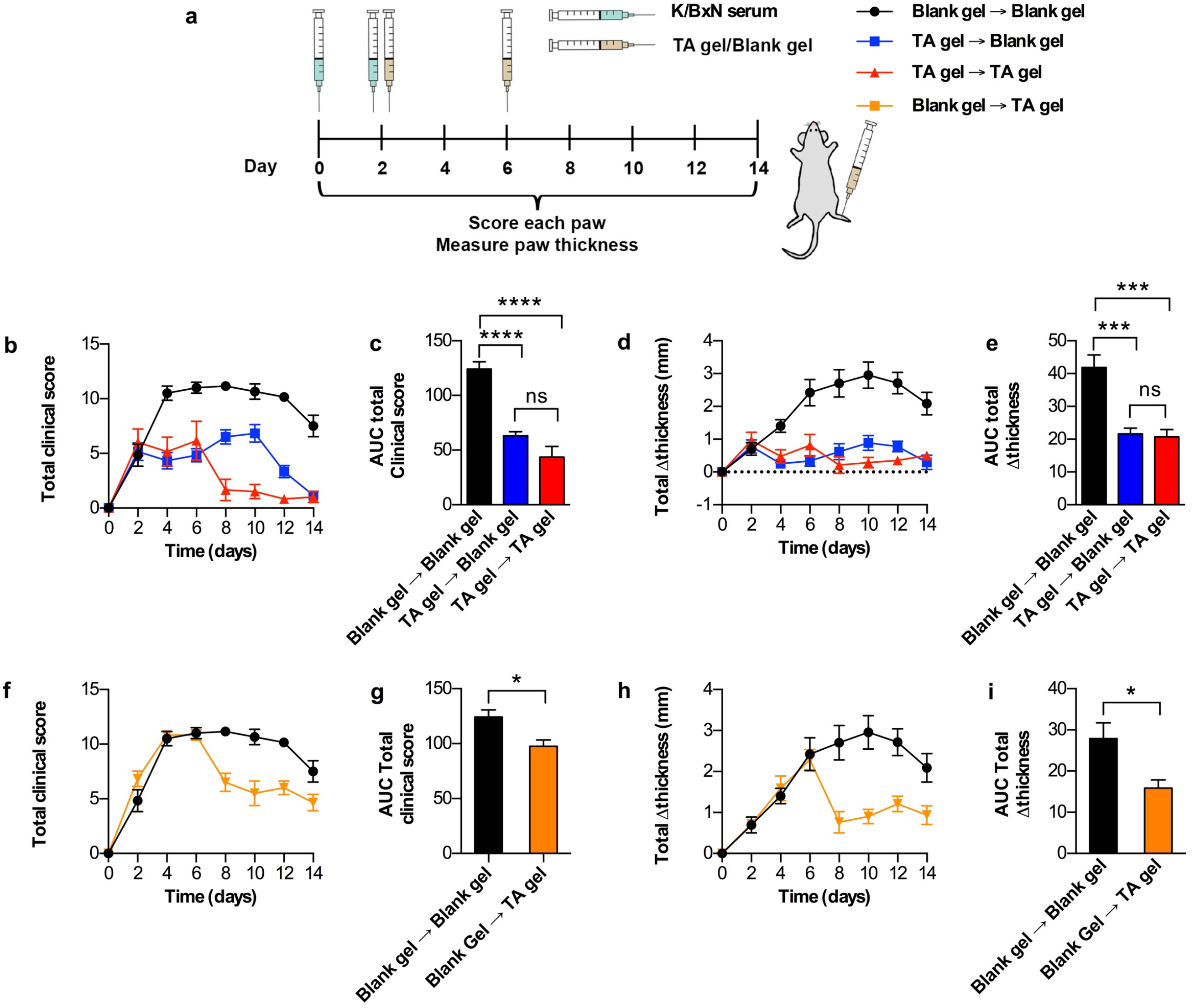

Supplementary Figure 8: Local delivery of TA loaded TG-18 hydrogel demonstrates therapeutic efficacy in severe arthritis: effect of dosing regimen. (a) Experimental outline: Arthritis was induced by i.p. injections of 75 µl KBx/N serum on day 0 and day 2. Immediately after the second dose of K/BxN serum, animals were randomized into four groups. (Blank gel → Blank gel) group received blank hydrogel (4 µl) on day 2 and 6, (TA gel → Blank gel) group received TA-loaded TG-18 hydrogel (20 mg TA/ml, 4 µl) on day 2 and blank hydrogel on day 6, (TA gel → TA gel) group received two doses of TA-loaded TG-18 hydrogel – one on day 2 and another on day 6 and (Blank gel → TA gel) group received blank hydrogel on day 2 and TA-loaded TG-18 hydrogel on day 6. Treatments were injected into the right hind paw. Every other day, arthritis severity was scored clinically and paw swelling was measured with calipers. **(b,c)** Total clinical score curves and their AUCs for (Blank gel → Blank gel), (TA gel → Blank gel) and (TA gel → TA gel) (*****P*<0.0001). **(d,e)** Change in

total paw thickness curves and their AUCs for (Blank gel → Blank gel), (TA gel → Blank gel) and (TA gel → TA gel) (****P*<0.001). **(f,g)** Total clinical score curves and their AUCs for (Blank gel → Blank gel), (TA gel → Blank gel) and (Blank gel → TA gel) (**P*<0.05). **(h,i)** Change in total paw thickness curves and their AUCs for (Blank gel → Blank gel), (TA gel → Blank gel) and (Blank gel → TA gel) (**P*<0.05). For (c,e), *P* values were determined by one-way Anova with Tukey's post hoc analysis and for (g,i), *P* values were determined by Student's *t* test. Data are means ± SEM (n = 6 mice per group).
